# Short-term and long-term efficacy of 7 targeted therapies for the treatment of advanced hepatocellular carcinoma: a network meta-analysis: Efficacy of 7 targeted therapies for AHCC

**DOI:** 10.1097/MD.0000000000005591

**Published:** 2016-12-09

**Authors:** Meng Niu, Duo Hong, Teng-Chuang Ma, Xiao-Wei Chen, Jin-Hang Han, Jun Sun, Ke Xu

**Affiliations:** Department of Radiology, First Hospital of China Medical University, Shenyang, P. R. China.

**Keywords:** advanced hepatocellular carcinoma, complete response, partial response, placebo, progressive disease, stable disease, targeted drug

## Abstract

**Background:**

A variety of targeted drug therapies in clinical trials have been proven to be effective for the treatment of hepatocellular carcinoma (HCC). Our study aims to compare the short-term and long-term efficacies of different targeted drugs in advanced hepatocellular carcinoma (AHCC) treatment using a network meta-analysis approach.

**Methods:**

PubMed, Embase, Ovid, EBSCO, and Cochrane central register of controlled trials were searched for randomized controlled trials (RCTs) of different targeted therapies implemented to patients with AHCC. And the retrieval resulted in 7 targeted drugs, namely, sorafenib, ramucirumab, everolimus, brivanib, tivantinib, sunitinib, and sorafenib+erlotinib. Direct and indirect evidence were combined to evaluate stable disease (SD), progressive disease (PD), complete response (CR), partial response (PR), disease control rate (DCR), overall response ratio (ORR), overall survival (OS), and surface under the cumulative ranking curve (SUCRA) of patients with AHCC.

**Results:**

A total of 11 RCTs were incorporated into our analysis, including 6594 patients with AHCC, among which 1619 patients received placebo treatment and 4975 cases had targeted therapies. The results revealed that in comparison with placebo, sorafenib, and ramucirumab displayed better short-term efficacy in terms of PR and ORR, and brivanib was better in ORR. Regarding long-term efficacy, sorafenib and sorafenib+erlotinib treatments exhibited longer OS. The data of cluster analysis showed that ramucirumab or sorafenib+erlotinib presented relatively better short-term efficacy for the treatment of AHCC.

**Conclusion:**

This network meta-analysis shows that ramucirumab and sorafenib+erlotinib may be the better targeted drugs for AHCC patients, and sorafenib+erlotinib achieved a better long-term efficacy.

## Abstract 2

Supplemental Digital Content is available in the text

## 1 Introduction

Hepatocellular carcinoma (HCC) is one of the most common clinical digestive malignant tumors,^[1]^ whose etiology has not been fully elucidated, yet the hepatitis, cirrhosis, and hepatic carcinoma are considered to be the 3 main reasons for its continued evolution of migration through long-term clinical observation.^[2–4]^ Other factors like alcohol and unhealthy living habits may also function as HCC inducement.^[5]^ Currently, multidisciplinary treatments of surgery, molecular targeted therapy, and traditional Chinese medicine are advocated for HCC treatment.^[6]^ Due to the insidious onset, high malignant degree, dissemination, and metastasis of HCC, the diagnosis of pathologically early HCC remains difficult.^[7]^ Therefore, it is common for majority HCC patients to develop advanced hepatocellular carcinoma (AHCC) at initial diagnosis and lose the opportunity of radical surgery and other local treatments.^[8,9]^ Therefore, it is of great importance to explore strategies for AHCC patients in order to further improve the overall efficacy of AHCC treatment.

In recent years, targeting drugs has gradually become a focus of HCC treatment, and there are a variety of targeted drug therapies in clinical trials that have been proven to be effective.^[10,11]^ Wherein, sorafenib, which is based on a category of multitargeted tyrosine kinase inhibitor, has been used in clinical trials for its wide range of antitumor effect.^[12]^ Sorafenib can effectively extend the overall survival time of HCC patients, but its severe side effects may affect the life quality of those patients.^[13]^ In addition to sorafenib, sunitinib, an oral multitargeted tyrosine kinase inhibitor, and brivanib, a selective dual inhibitor of vascular endothelial growth factor (VEGF) and fibroblast growth factor signaling, are the most concerned agents targeted to AHCC management with effective outcomes;^[1]^ whereas, tivantinib, a receptor tyrosine kinase encoded from a proto-oncogene c-Met gene, can cause cell death by acting on the caspase-dependent apoptosis pathway.^[14]^

In addition to tyrosine kinase inhibitors, everolimus, an oral small-molecule serine-threonine kinase inhibitor, demonstrates a good drug resistance of AHCC with fewer adverse reactions though inhibition of certain signaling pathway.^[15]^ Ramucirumab also displays satisfactory clinical results as well for its angiogenesis inhibition of tumor.^[16]^ Currently, clinical assessment shows that sunitinib possess superior effects than sorafenib, which may represent a new generation of targeted regimen.^[17]^ Considering the unsatisfactory results in angiogenesis of HCC, erlotinib has also been reported as a promising target for HCC owing to its ability to inhibit phosphorylation of the intracellular domain of the epidermal growth factor receptor (EGFR).^[18]^ The comparisons on efficacy among different drug treatments cannot be achieved through traditional meta-analysis, but can be accomplished depending on network meta-analysis, which implements a quantified comparison with similar disease interventions for the selection of the optimal treatment strategy.^[19]^ Therefore, this study enrolled 11 randomized controlled trials (RCTs) based upon a network meta-analysis to evaluate the efficacies of 7 targeted drugs, including sorafenib, ramucirumab, everolimus, brivanib, tivantinib, sunitinib, and sorafenib + erlotinib with the expectation to provide supporting evidence for a reasonable choice for AHCC treatment.

## 2 Materials and methods

### 2.1 Ethics statement

Our study is a network meta-analysis and the ethics statement is not applicable.

### 2.2 Literature search

PubMed, Embase, Cochrane central register of controlled trials, Ovid, EBSCO, and other English databases were searched from the inception of each database to September 2016. The search was conducted using MeSH terms, keywords, and combined words, which include: liver neoplasms, cancer of liver, hepatocellular cancer, hepatic cancer, sorafenib, metuximab, trastuzumab, ramucirumab, cetuximab, matuzumab, panitumumab, sunitinib, everolimus, brivanib, temsirolimus, celecoxib, lapatinib, randomized controlled trial, and so on.

### 2.3 Inclusion and exclusion criteria

The inclusion criteria: study design – RCT; interventions – targeted drug/placebo for AHCC patients; study subject – patients with AHCC; and end outcomes – stable disease (SD), progressive disease (PD), complete response (CR), partial response (PR), disease control rate (DCR), overall response ratio (ORR), and overall survival (OS). The exclusion criteria: studies with insufficient data; non-RCTs; non-AHCC; non-English reference; and duplicated publications by the same author using the same interventions.

### 2.4 Data extraction and quality assessment

Two reviewers extracted data from the enrolled studies using a specifically designed form. Additionally, a 3rd reviewer was consulted if those 2 reviewers failed to reach an agreement. Researchers of 2 or more reviewed the RCTs according to Cochrane risk of bias assessment tools,^[20]^ which include 6 domains, namely, random allocation, allocation concealment, blinding, loss outcome data, selected outcome reports, and other bias. The assessment included a judgment assignment of “yes,” “no,” or “unclear” for each domain to designate a low, high, or unclear risk of bias. The study was classified as a low risk of bias if one or no domain was deemed “unclear” or “no,” a high risk of bias if 4 or more domains are deemed “unclear” or “no” and a moderate risk of bias if 2 or 3 domains were deemed “unclear” or “no.”^[21]^ Quality assessment and investigation of publication bias were conducted using Review Manager 5 (RevMan 5.2.3, Cochrane Collaboration, Oxford, UK.

### 2.5 Statistical analysis

Traditional pairwise meta-analyses were performed to directly compare different treatment arms. Then Bayesian network meta-analyses were applied for direct comparisons of different interventions to each other. The results were reported as odds ratios (ORs) or hazard ratio (HR) with 95% CI accounting for study sample sizes. The node-splitting plot statistic was conducted to assess the extent of inconsistency, and ontology consistent model was applied if *P* > 0.05. Each analysis was grounded on noninformative priors to obtain effect sizes and precision. Convergence and lack of auto correlation were examined and confirmed after 4 chains and a 20,000-simulation burn-in phase. Subsequently, direct probability statements were derived from an additional 50,000-simulation phase.^[22]^ Surface under the cumulative ranking curve (SUCRA) was applied to provide a ranking among the included treatments and indicate which treatment was the optimal one.^[23]^ The network plot of interventions was a representation of the evidence base and conferred a concise description of its characteristics.^[24]^ Cluster analyses were used to group the treatments regarding their similarity on outcomes.^[25]^ All computations were performed using R (V.3.1.2) package gemtc (V.0.6), 13 14, and the Markov Chain Monte Carlo engine Open BUGS (V.3.4.0).

## 3 Results

### 3.1 Baseline characteristics

**Figure 1:**
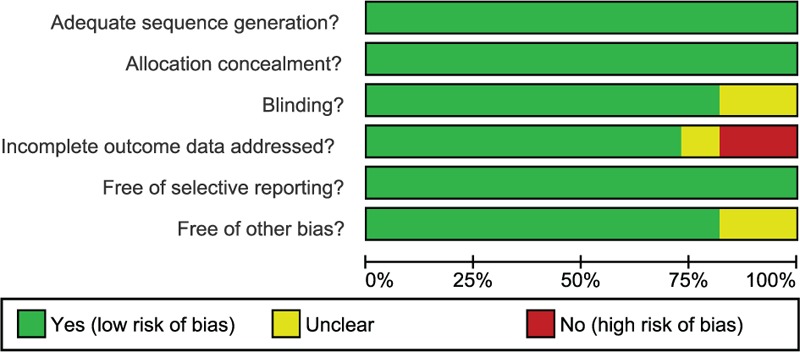
Quality assessment for all included studies using Cochrane systematic bias.

Aforementioned electronic database retrieve delivered 1364 eligible studies. After reviewing the titles and abstracts, 183 studies were excluded as duplicates, 95 as letters or summarizations, 31 as non-English studies, 569 as unrelated to AHCC, and 324 as nontargeted drug studies. Based upon further evaluation of the remaining 162 articles, 69 studies of nonrandomized study were removed, along with 82 studies without data resources or incomplete documentations. Eventually, 11 RCTs were considered eligible for this network meta-analysis^[26–36]^ (Fig. S1), which, altogether, included 6594 cases of patients with AHCC with 1619 cases were treated with placebo treatment (8 studies), 2526 cases were treated with sorafenib treatment (7 studies), 283 cases were treated with ramucirumab treatment (1 study), 362 cases were treated with everolimus treatment (1 study), 841 cases were treated with brivanib treatment (2 studies), 71 patients were treated using tivantinib (1 study), 530 cases were treated with sunitinib treatment (1 study), and 362 cases were treated with sorafenib + erlotinib treatment (1 study). Published during 2008 to 2015, all the 11 studies were 2-arm trials, whose baseline characteristics were presented in Table S1 and Cochrane systematic bias were shown in Fig. 1.

### 3.2 Pairwise meta-analysis

Direct paired comparisons for efficacies of the 8 treatments of AHCC were conducted, suggesting better efficacies of ramucirumab and tivantinib than that of placebo in terms of SD (ramucirumab: OR = 1.59, 95%CI = 1.18–2.14; tivantinib: OR = 1.52, 95%CI = 1.06–2.17). Sorafenib's efficacy was relatively better regarding SD compared with sorafenib + erlotinib treatments (OR = 3.07, 95%CI = 1.69–5.58, Fig. S2A). Everolimus and brivanib exhibited better efficacies than that of placebo as for PD (everolimus: OR = 0.62, 95%CI = 0.44–0.87; brivanib: OR = 0.44, 95%CI = 0.19–1.00), and there was no distinctive difference on efficacies of sorafenib, ramucirumab, and tivantinib with placebo. In comparison with sorafenib + erlotinib, the effect of sorafenib was superior (OR = 0.38, 95%CI = 0.21–0.67), and no significant difference was revealed between brivanib and sorafenib (Fig. S2B). In terms of CR, there was no significant difference on effects of sorafenib, ramucirumab, everolimus, brivanib, and tivantinib compared to placebo, so was the efficacy of sorafenib to sorafenib + erlotinib, and brivanib, sunitinib, and sorafenib to sorafenib (Fig. S2C). The efficacy of everolimus was more satisfactory in comparison with placebo considering PR (OR = 10.08, 95%CI = 2.32–43.68), and there was no significant difference in efficacies of sorafenib, ramucirumab, brivanib, and tivantinib compared to placebo, so was the efficacy of sorafenib to sorafenib + erlotinib, and brivanib and sunitinib to sorafenib (Fig. S2D). The efficacies of ramucirumab, everolimus, and tivantinib were more favorable in terms of DCR compared to placebo (ramucirumab: OR = 1.41, 95%CI = 1.05–1.89; everolimus: OR = 1.52, 95%CI = 1.09–2.12; tivantinib: OR = 1.55, 95%CI = 1.09–2.22), and the efficacy of sorafenib and brivanib demonstrated no significant difference compared to placebo. Sorafenib was found to present superior efficacy compared with sorafenib + erlotinib (OR = 3.30, 95%CI = 1.82–5.96), and no significant difference was indicated between the effects of brivanib and sunitinib and that of sorafenib (Fig. S2E). Everolimus was more efficient in terms of ORR in comparison with placebo (OR = 10.65, 95%CI = 2.46–45.99, Fig. S2F). Sorafenib indicated a longer OS than placebo (HR = 0.69, 95%CI = 0.60–0.79, Fig. S3).

### 3.3 Pooled results of network meta-analysis

#### 3.3.1 Network Plot

**Figure 2:**
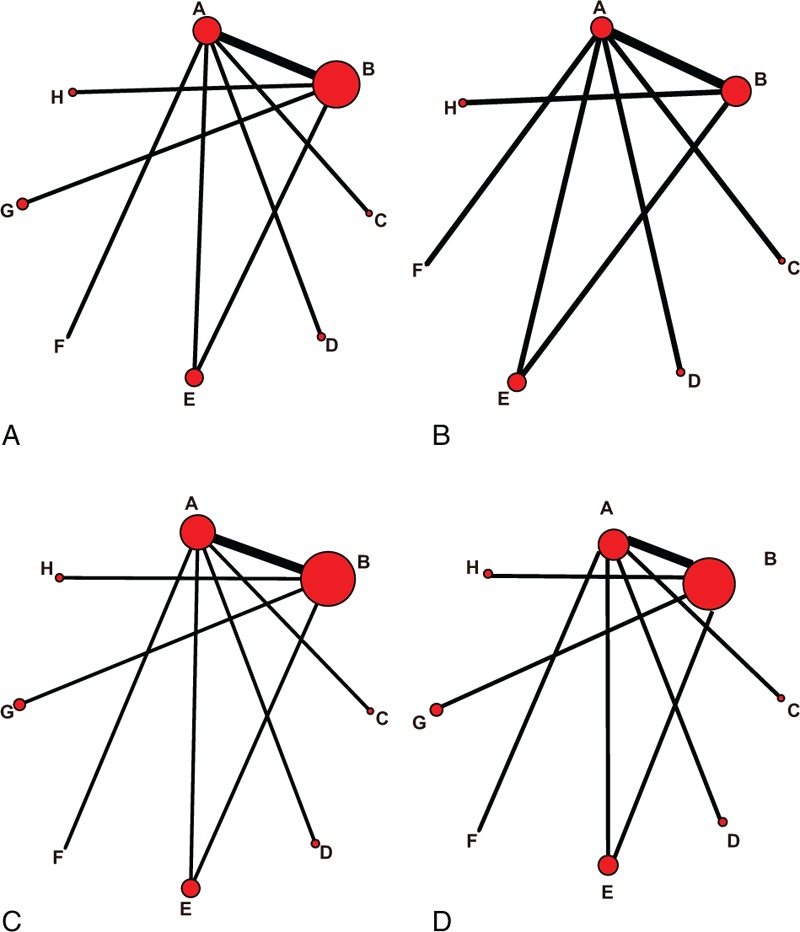
Network plot of the 7 targeted drugs. Nodes and lines are weighted according to the number of studies providing direct comparisons between 2 treatments. Nodes are weighted according to the number of studies including the respective interventions. Edges are weighted according to the inverse of variance. ([A] SD and DCR; [B] PD; [C] CR, PR and ORR; [D] OS; A: placebo; B: sorafenib; C: ramucirumab; D: everolimus; E: brivanib; F: tivantinib; G: sunitinib; H: sorafenib + erlotinib; ORR: CR + PR; DCR = SD + CR + PR). CR = complete response, DCR = disease control rate, OR = odds ratio, ORR = overall response ratio, OS = overall survival, PD = progressive disease, PR = partial response, SD = stable disease.

A total of 7 kinds of targeted drugs (sorafenib, ramucirumab, everolimus, brivanib, tivantinib, sunitinib, sorafenib + erlotinib) and placebo, were included in this study. Node size in the network plot represented the number of the included subjects, and the width of nodes and lines indicated the accuracy of effect size (the inverse of variance). The network plot of SD and DCR were shown in Fig. 2A, PD in Fig. 2B, CR, PR, and ORR in Fig. 2C, and OS in Fig. 2D.

#### 3.3.2 Inconsistency test

**Figure 3:**
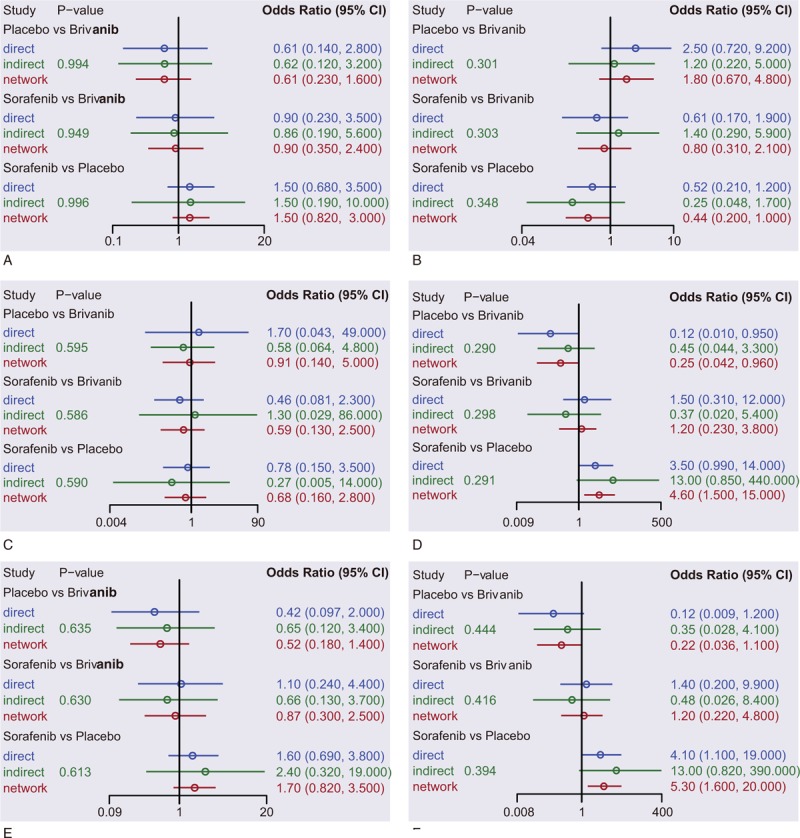
Node space (A) stable disease; (B) progressive disease; (C) complete response; (D) partial response; (E) disease control rate; and (F) overall response ratio; overall response ratio: complete response + partial response; disease control rate = stable disease + overall response ratio.

**Figure 4:**
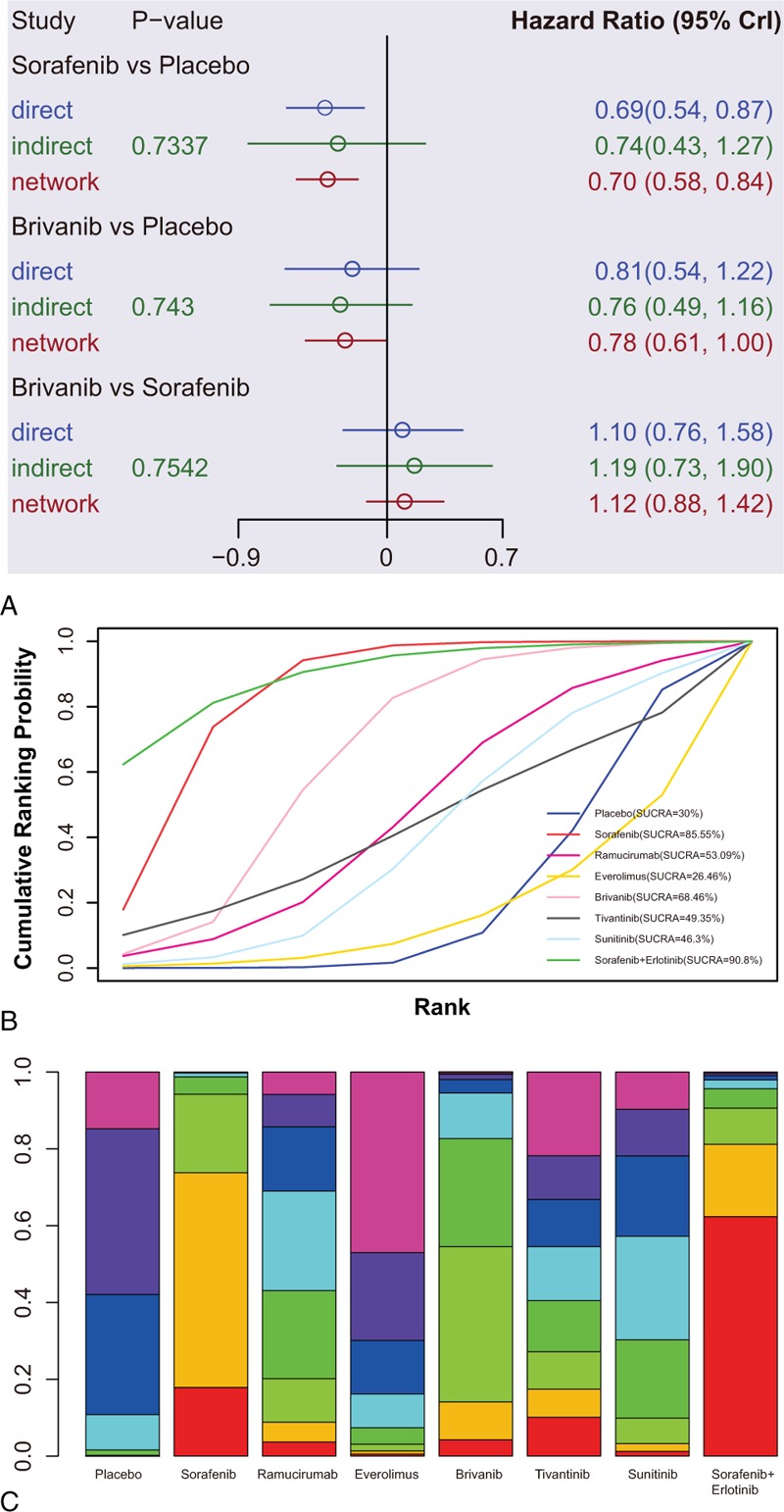
Node space, SUCRA curves and ranking plot of targeted drug effects on long-term efficacy of AHCC. (A) Node space; (B) SUCRA curves; and (C) ranking plot.

Six studies among the total 11 constituted a closed loop to implement the inconsistency test (sorafenib vs placebo: 4 studies; brivanib vs placebo: 1 study; and brivanib vs sorafenib: 1 study), which showed no inconsistencies among all the studies in terms of SD, PD, CR, PR, DCR, ORR, and OS (all *P* > 0.05) (Fig. 3A–F, Fig. 4A). Therefore, consistency model was applied.

#### 3.3.3 Relative relationship in short-term efficacy of AHCC

The relative relationship on direct and indirect short-term efficacy of the 8 treatments for patients with AHCC showed that: there was no significant difference among all the treatments in terms of SD, PD, CR, and DCR. Regarding PR, the efficacies of sorafenib and ramucirumab were better than that of placebo (sorafenib: OR = 4.82, 95%CI = 1.37–15.91; ramucirumab: OR = 12.26, 95%CI = 1.36–159.79), while no significant difference was revealed among all the treatments. In terms of ORR, the efficacies of sorafenib, ramucirumab, and brivanib were superior compared to the efficacy of placebo (sorafenib: OR = 5.23, 95%CI = 1.60–18.73; ramucirumab: OR = 13.97, 95%CI = 1.33–160.57; and brivanib: OR = 4.41, 95%CI = 1.08–27.16), and no significant difference was presented among all the regiments. The treatments of sorafenib and sorafenib + erlotinib exhibited better prognosis in the aspect of OS in comparison with placebo (Table 1, Table S2).

**Table 1:**
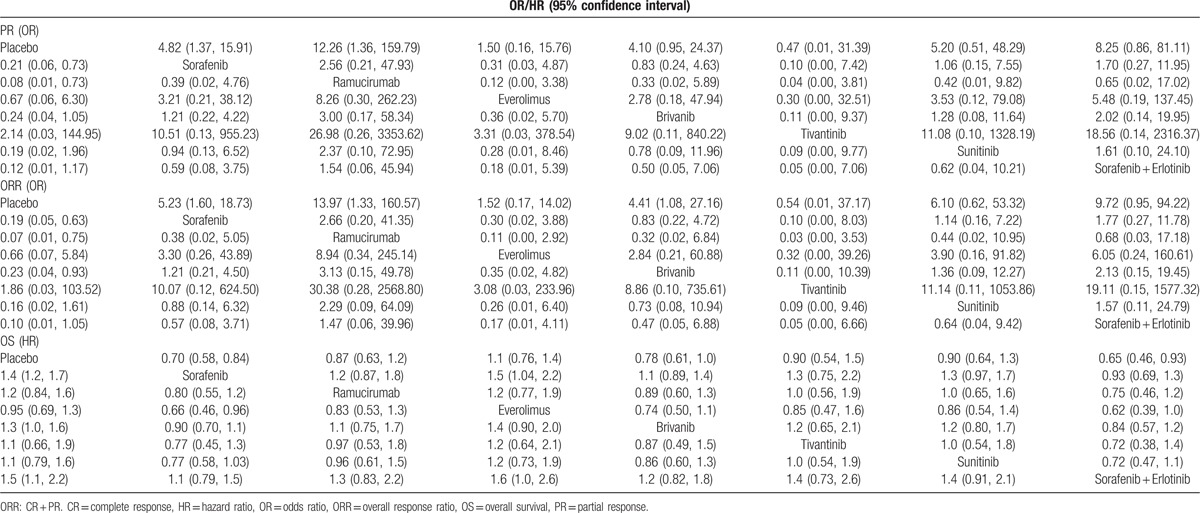
ORs or HR and 95% confidence intervals of 8 treatment modalities under 3 end indicators according to the network meta-analysis.

#### 3.3.4 SUCRA

**Figure 5:**
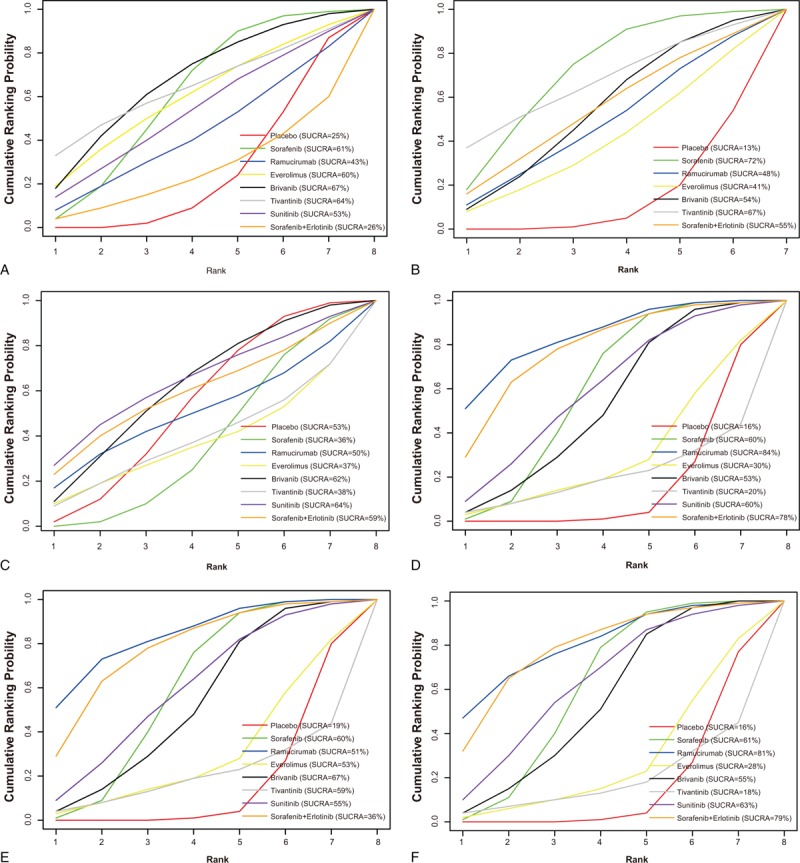
SUCRA curves for all treatments under outcomes (A) stable disease; (B) progressive disease; (C) complete response; (D) partial response; (E) disease control rate; and (F) overall response ratio; overall response ratio: complete response + partial response; disease control rate = stable disease + overall response ratio.

**Figure 6:**
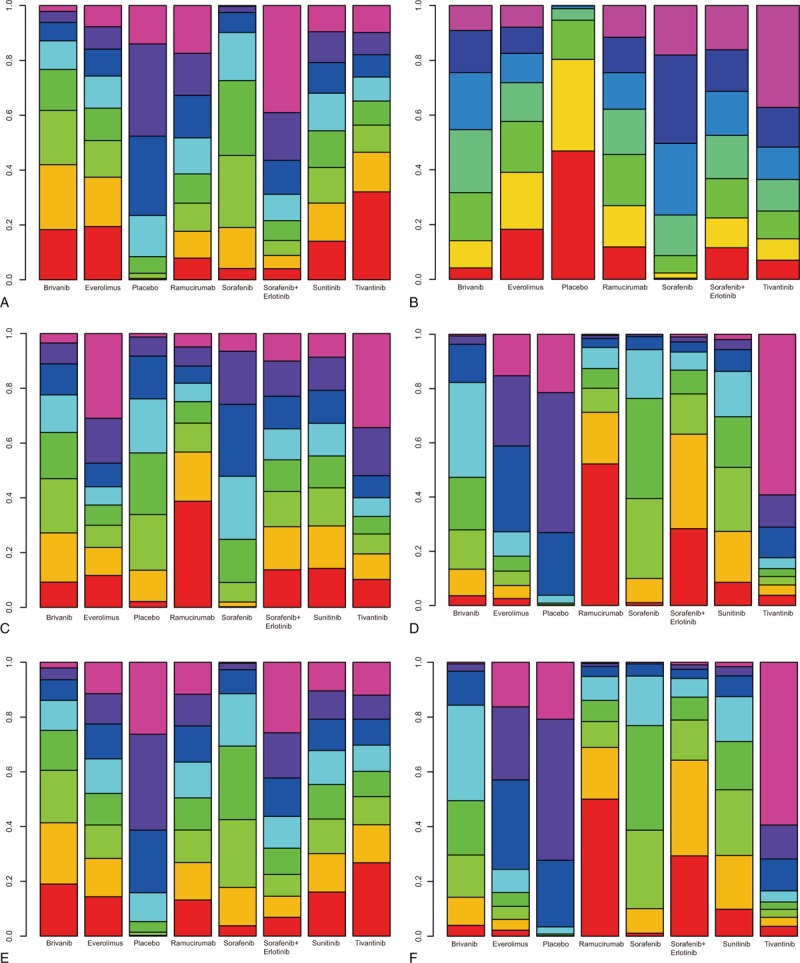
Ranking plot for all treatments under outcomes (A) stable disease; (B) progressive disease; (C) complete response; (D) partial response; (E) disease control rate; and (F) overall response ratio; overall response ratio: complete response + partial response; disease control rate = stable disease + overall response ratio.

SUCRA values of the 8 treatments of AHCC patients were described in the Table 2. Detailed SUCRA curve under the 6 outcome indicators was presented in Fig. 4B and Fig. 5A–F. Sorted results of SUCRA values showed that brivanib was the optimal targeted drug for the treatment of AHCC in terms of SD and DCR, whose SUCRA values were 0.67 and 0.67, respectively. Sorafenib was the best targeted drug in terms of PD with the SUCRA value of 0.72. Sunitinib claimed the best efficacy in view of CR, whose SUCRA value was 0.64. Ramucirumab achieved the best efficacy considering PR and ORR, whose SUCRA values were 0.84 and 0.81, respectively. The ranking plot results suggested that tivantinib presented the highest probability to rank 1st in the cumulative probability of SD, PD, and DCR, while ramucirumab exhibited the highest probability to occupy the 1st place in the cumulative probability of CR, PR, and ORR (Fig. 4B and Fig. 6A–F).

**Table 2:**
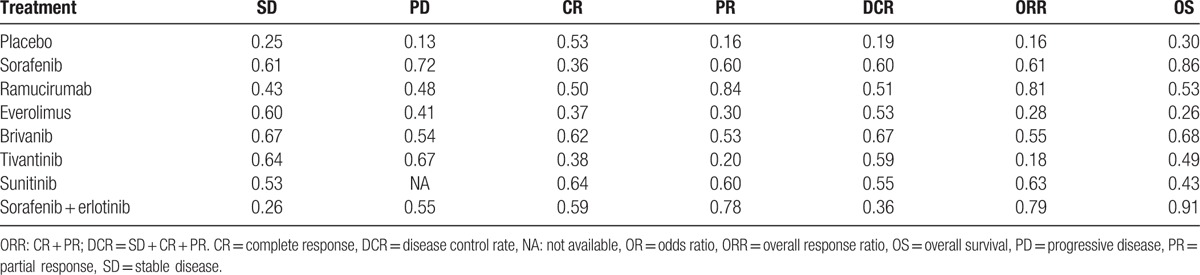
The results of surface under the cumulative ranking curve of 8 treatment modalities.

#### 3.3.5 Cluster analysis

**Figure 7:**
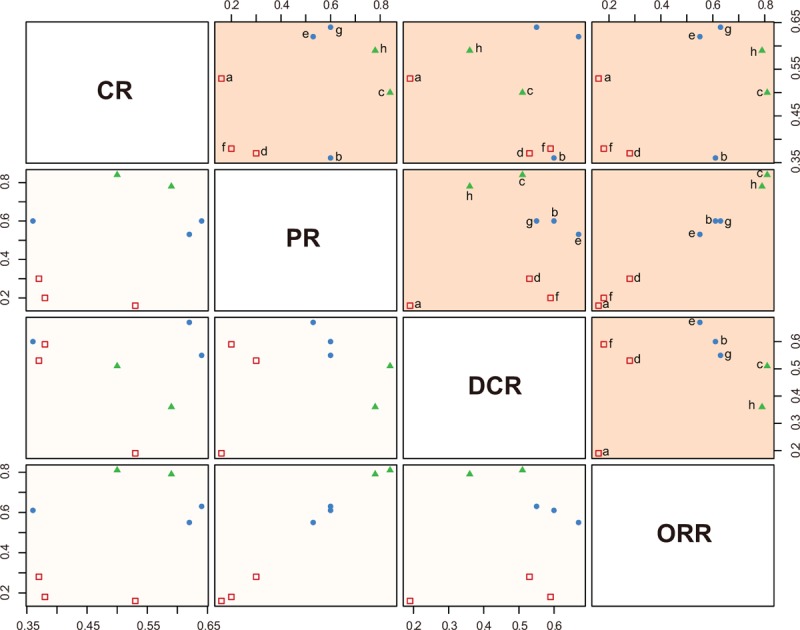
Clustered ranking plots based on SUCRA values of 4 outcomes. (a: placebo; b: sorafenib; c: ramucirumab; d: everolimus; e: brivanib; f: tivantinib; g: sunitinib; h: sorafenib + erlotinib; CR; PR; DCR; ORR; ORR: CR + PR; DCR = SD + CR + PR). CR = complete response, DCR = disease control rate, ORR = overall response ratio, PD = progressive disease, PR = partial response, SD = stable disease.

Results of cluster analysis of SUCRA values based on CR versus PR, CR versus DCR, and CR versus ORR indicated that the short-term efficacies of ramucirumab and sorafenib + erlotinib for patients with AHCC were more favorable, and the placebo, everolimus, and tivantinib treatment delivered a poorer short-term efficacy. Results of cluster analysis of SUCRA value based on PR versus DCR, and PR versus ORR revealed that the efficacies of ramucirumab and sorafenib + erlotinib were better, and the placebo, everolimus, and tivantinib presented a relatively dissatisfactory efficacy. Based on DCR versus ORR, it is indicated that the short-term efficacies of ramucirumab and sorafenib + erlotinib were superior, and the placebo, everolimus, and tivantinib treatment were inferior in short-term efficacy. Cluster analysis was not applied for long-term efficacy, because there was only 1 outcome indicator OS for the long-term effects (Fig. 7).

## 4 Discussion

Network meta-analysis was applied to evaluate the efficacy of 7 kinds of targeted drugs for AHCC treatment, namely, sorafenib, ramucirumab, everolimus, brivanib, tivantinib, sunitinib, and sorafenib + erlotinib, which have been widely discussed in HCC.^[10]^ Our results indicated that ramucirumab and sorafenib + erlotinib were superior targeted drugs for AHCC management in terms of short-term effects, and sorafenib + erlotinib exhibited better efficacy in the long term.

Initially, our analysis revealed that ramucirumab and sorafenib + erlotinib presented more satisfactory results in the short-term efficacy concerning PR and ORR, and brivanib was better in terms of ORR. Ramucirumab has been approved by the Food and Drug Administration to be used for the treatment of metastatic nonsmall cell lung carcinoma.^[37]^ Ramucirumab may be effective in AHCC treatment by contributing to angiogenesis which plays an important role in the invasion and metastasis of solid tumor and in the neovascularization based on VEGF and vascular endothelial growth factor receptor (VEGFR).^[16,38,39]^ As a kind of VEGFR2 antagonists, ramucirumab is capable of specifically binding VEGFR2 extracellular domain, which can prevent the binding of VEGFR2 with its ligand and thereby inhibit the activation of VEGFR2 and the conduction of downstream signaling pathways.^[40]^ Thus, angiogenesis is prevented to block blood supply to tumor cells, which leads to tumor cell apoptosis.^[41]^ Ueda et al confirm the efficacy of ramucirumab in advanced gastric adenocarcinomas achieving best overall responses with SD (n = 5) and PR (n = 1).^[42]^

Quintela-Fandino et al^[43]^ demonstrate in phase I clinical trial of HCC that the combination of sorafenib + erlotinib can inhibit the tumor cell proliferation in a more efficient fashion. The possible explanation for this result might be that as a small molecule tyrosine ammonia kinase inhibitor, erlotinib can prevent the autophosphorylation of EGFR.^[18]^ The inhibition of cell proliferation, angiogenesis, invasion, and metastasis and the inducement of apoptosis eventually can exert antitumor effects.^[44]^ The experiment of Gu et al^[45]^ indicates that the inhibition of tumor growth and metastasis of sorafenib is achieved by blocking mitogen-activated protein kinases/extracellular signal-regulated kinases/signal transducer and activator of transcription 3 and phosphatidylinositol-3-kinase/protein kinase B/signal transducer and activator of transcription 3 signaling pathways, both of which are different in tumor suppression mechanism which plays a synergistic effect in inhibition of AHCC. Another possible explanation revealed that sorafenib + erlotinib can inhibit the tumor growth mainly in the process of synergy in G0/G1 phase, and meanwhile activate caspase3 and regulate the balance of antiapoptotic molecule B-cell leukemia/lymphoma 2 and pro-apoptotic molecules B-cell leukemia/lymphoma 2-associated X protein,^[46]^ thus increasing the inhibitory effect of drug combination on tumor treatment. The experiment of Lind et al^[47]^ indicated the efficacy of erlotinib and sorafenib for the treatment of chemotherapy-naive patients with advanced nonsmall cell lung cancer of 24% PR, 50% SD, and ultimately ORR of 28%. Brivanib functions as a dual inhibitor of VEGF receptor tyrosine kinases,^[48]^ which demonstrates potent antiproliferative and antiangiogenic effects on various tumor cells, including hepatoma cells.^[49,50]^ Llovet et al^[30]^ reported an ORR of 10% for brivanib based on modified response evaluation criteria in solid tumors compared to 2% for placebo.

Furthermore, it is indicated in our study that sorafenib + erlotinib presented longer OS in the aspect of long-term efficacy. In phase II trial of unselected patients with pretreated advanced nonsmall cell lung cancer, sorafenib as single agent has conferred a promising antitumor effect, with a median OS of 6.7 months and a median progression-free survival of 2.7 months with an acceptable toxicity.^[51]^ Sorafenib is the only systemic treatment to indicate a significant but modest OS benefit, resulting in an era of targeted drugs.^[10]^ Gridelli et al^[52]^ demonstrate that combined erlotinib and sorafenib is feasible in elderly patients with advanced nonsmall cell lung cancer and is correlated with a higher 1-year survival rate than the other arm. It is also suggested by Spigel et al^[53]^ that EGFR-negative patients reveal a benefit for the combination of erlotinib and sorafenib compared with erlotinib alone in terms of progression-free survival and OS,^[53]^ which is consistent with our findings.

The advantages and disadvantages of different drug efficacy can be achieved through network meta-analysis method, whereby different treatments with similar disease interventions can be implemented with a quantified comparison and thus confers optimal treatment.^[19]^ However, this study has some limitations. First, the included 11 documents are limited in number; 2nd, limited targeted drug treatments may result in selection bias, and insufficient emphasis on the allocation concealment may exaggerate the effects of treatment; ultimately, the included studies fail to indicate the reasons for patients who were lost during follow-up, which may affect the evaluation of the therapeutic effect.

The results of network meta-analysis in this study suggested that the short-term efficacy of ramucirumab and sorafenib + erlotinib for AHCC patients were superior to other drugs, and sorafenib + erlotinib was better in long-term efficacy, which may shed a little light for the clinical treatment of AHCC. However, the limitation of network meta-analysis requires further studies to comprehensively evaluate the efficacy of different targeted drugs taking overall outcomes of patients, survival rate, and cost-effectiveness into consideration.

## Acknowledgments

The authors thank the reviewers for their helpful comments on this paper.

## Supplementary Material

Supplemental Digital Content

## Supplementary Material

Supplemental Digital Content

## Supplementary Material

Supplemental Digital Content

## Supplementary Material

Supplemental Digital Content
