# Supplementary material for: Short-term and long-term efficacy of 7 targeted therapies for the treatment of advanced hepatocellular carcinoma: a network meta-analysis: Efficacy of 7 targeted therapies for AHCC
Source: Medicine (Baltimore). 2016 Dec 9;95(49):e5591. doi: 10.1097/MD.0000000000005591 (PMC5266050; doi:10.1097/MD.0000000000005591)
Supplement: Supplemental Digital Content [file medi-95-e5591-s001.docx]

**Table S1.** Main baseline characteristics and methodological quality of eligibly studies included in this network meta-analysis.

| **Author** | **Year** | **Country** | **Patients numbers** | **Targeted drugs** | | **Study Design** | **Follow-up**  **(months)** | **Gender(M/F)** | | **Age(years)** | |
| --- | --- | --- | --- | --- | --- | --- | --- | --- | --- | --- | --- |
|  |  |  |  | **Treat 1** | **Treat 2** |  |  | **Treat 1** | **Treat 2** | **Treat 1** | **Treat 2** |
| Zhu AX-a | 2015 | USA | 720 | Sorafenib | Sorafenib+Erlotinib | RCT | 0-32 | 286/72 | 295/67 | 0-32 | 60.5 |
| Zhu AX-b | 2015 | USA | 565 | Ramucirumab | Placebo | RCT | 8.3(4.0-14.9) | 236/47 | 242/40 | 8.3(4.0-14.9) | 62(25-85) |
| Zhu AX | 2014 | USA | 546 | Everolimus | Placebo | RCT | 24.6(14.8-36.6) | 303/59 | 160/24 | 24.6(14.8-36.6) | 64(34-87) |
| Santoro A | 2013 | Belgium | 107 | Tivantinib | Placebo | RCT | 5.5(0.6-22.0) | 58/13 | 28/8 | 5.5(0.6-22.0) | 68(46-85) |
| Llovet JM | 2013 | Spain | 395 | Brivanib | Placebo | RCT | 0-30 | 216/47 | 113/19 | 0-30 | 62(19-87) |
| Johnson PJ | 2013 | USA | 1155 | Brivanib | Sorafenib | RCT | 0-35 | 484/94 | 483/94 | 0-35 | 61(19-87) |
| Cheng AL | 2013 | China | 1074 | Sunitinib | Sorafenib | RCT | 0-30 | 436/94 | 459/85 | 0-30 | 59(18-84) |
| Kane RC | 2009 | USA | 602 | Sorafenib | Placebo | RCT | 0-17 | 523/79 | | 0-17 | |
| Cheng AL | 2009 | China | 226 | Sorafenib | Placebo | RCT | 0-20 | 127/23 | 66/10 | 51(23-86) | 52(25-79) |
| Zhu AX | 2008 | China | 602 | Sorafenib | Placebo | RCT | NR | NR NR | | NR NR | |
| Llovet JM | 2008 | Spain | 602 | Sorafenib | Placebo | RCT | 0-17 | 260/39 | 264/39 | 0-17 | 66.3 ± 10.2 |

Notes: RCT: Randomized controlled trials; NR: Not reported; M: Male; F: Female; a: Sorafenib+Erlotinib vs. Sorafenib; b: Ramucirumab vs. Placebo.

**Table S2.** Odds ratio and 95% confidence intervals of eight treatment modalities under four end indicators according to the network meta-analysis.

| **OR(95% confidence interval)** | | | | | | | |
| --- | --- | --- | --- | --- | --- | --- | --- |
| **SD** |  |  |  |  |  |  |  |
| Placebo | 1.48 (0.81, 3.08) | 1.18 (0.35, 4.11) | 1.51 (0.43, 5.35) | 1.63 (0.65, 4.44) | 1.66 (0.38, 7.35) | 1.38 (0.36, 6.00) | 0.92 (0.25, 4.06) |
| 0.67 (0.32, 1.23) | Sorafenib | 0.79 (0.18, 3.21) | 1.03 (0.23, 3.87) | 1.11 (0.42, 2.77) | 1.11 (0.21, 5.30) | 0.93 (0.27, 3.13) | 0.62 (0.18, 2.21) |
| 0.85 (0.24, 2.87) | 1.26 (0.31, 5.47) | Ramucirumab | 1.30 (0.21, 7.66) | 1.39 (0.29, 6.98) | 1.41 (0.21, 9.31) | 1.16 (0.19, 8.49) | 0.79 (0.13, 5.77) |
| 0.66 (0.19, 2.30) | 0.97 (0.26, 4.30) | 0.77 (0.13, 4.80) | Everolimus | 1.08 (0.24, 5.34) | 1.10 (0.16, 7.83) | 0.91 (0.15, 6.37) | 0.61 (0.10, 4.38) |
| 0.61 (0.23, 1.54) | 0.90 (0.36, 2.39) | 0.72 (0.14, 3.50) | 0.93 (0.19, 4.17) | Brivanib | 1.01 (0.18, 5.55) | 0.84 (0.19, 4.06) | 0.56 (0.13, 2.85) |
| 0.60 (0.14, 2.64) | 0.90 (0.19, 4.78) | 0.71 (0.11, 4.76) | 0.91 (0.13, 6.24) | 0.99 (0.18, 5.57) | Tivantinib | 0.83 (0.11, 6.55) | 0.56 (0.08, 4.61) |
| 0.73 (0.17, 2.77) | 1.07 (0.32, 3.70) | 0.86 (0.12, 5.40) | 1.10 (0.16, 6.77) | 1.19 (0.25, 5.36) | 1.20 (0.15, 8.98) | Sunitinib | 0.67 (0.12, 3.98) |
| 1.09 (0.25, 4.01) | 1.60 (0.45, 5.41) | 1.27 (0.17, 7.95) | 1.64 (0.23, 9.74) | 1.78 (0.35, 7.91) | 1.77 (0.22, 13.09) | 1.50 (0.25, 8.54) | Sorafenib+Erlotinib |
| **PD** |  |  |  |  |  |  |  |
| Placebo | 0.45 (0.20, 1.01) | 0.62 (0.18, 2.18) | 0.71 (0.21, 2.47) | 0.56 (0.20, 1.44) | 0.43 (0.10, 1.80) | 0.56 (0.13, 2.35) |  |
| 2.24 (0.99, 4.99) | Sorafenib | 1.38 (0.32, 6.16) | 1.57 (0.37, 6.92) | 1.25 (0.47, 3.15) | 0.96 (0.19, 5.04) | 1.23 (0.36, 4.15) |  |
| 1.60 (0.46, 5.62) | 0.72 (0.16, 3.15) | Ramucirumab | 1.15 (0.20, 6.45) | 0.90 (0.17, 4.21) | 0.68 (0.10, 4.79) | 0.90 (0.13, 5.59) |  |
| 1.41 (0.40, 4.82) | 0.64 (0.14, 2.72) | 0.87 (0.16, 4.90) | Everolimus | 0.79 (0.16, 3.74) | 0.60 (0.09, 4.03) | 0.79 (0.11, 5.05) |  |
| 1.79 (0.69, 4.93) | 0.80 (0.32, 2.14) | 1.11 (0.24, 5.81) | 1.27 (0.27, 6.21) | Brivanib | 0.78 (0.14, 4.57) | 0.99 (0.23, 4.78) |  |
| 2.34 (0.55, 9.73) | 1.04 (0.20, 5.30) | 1.46 (0.21, 9.61) | 1.66 (0.25, 10.86) | 1.28 (0.22, 7.20) | Tivantinib | 1.28 (0.17, 9.68) |  |
| 1.79 (0.42, 7.73) | 0.81 (0.24, 2.75) | 1.11 (0.18, 7.52) | 1.26 (0.20, 8.71) | 1.01 (0.21, 4.38) | 0.78 (0.10, 5.93) | Sorafenib+Erlotinib |  |
| **CR** |  |  |  |  |  |  |  |
| Placebo | 0.66 (0.14, 2.57) | 1.82 (0.15, 47.45) | 0.54 (0.01, 18.75) | 1.11 (0.17, 6.99) | 0.48 (0.01, 14.10) | 1.06 (0.10, 11.98) | 1.00 (0.08, 14.98) |
| 1.51 (0.39, 6.92) | Sorafenib | 2.95 (0.13, 84.61) | 0.78 (0.01, 44.42) | 1.63 (0.39, 8.05) | 0.77 (0.01, 27.74) | 1.62 (0.22, 12.50) | 1.52 (0.17, 16.68) |
| 0.55 (0.02, 6.88) | 0.34 (0.01, 7.79) | Ramucirumab | 0.30 (0.00, 19.08) | 0.57 (0.02, 15.12) | 0.24 (0.00, 14.49) | 0.51 (0.01, 22.01) | 0.52 (0.01, 21.24) |
| 1.86 (0.05, 77.79) | 1.28 (0.02, 68.51) | 3.37 (0.05, 611.18) | Everolimus | 2.02 (0.03, 159.03) | 0.91 (0.01, 134.67) | 2.07 (0.02, 182.49) | 2.08 (0.02, 187.69) |
| 0.90 (0.14, 5.96) | 0.61 (0.12, 2.58) | 1.74 (0.07, 54.18) | 0.50 (0.01, 34.79) | Brivanib | 0.47 (0.01, 18.90) | 0.97 (0.08, 13.43) | 0.96 (0.06, 14.04) |
| 2.08 (0.07, 88.99) | 1.30 (0.04, 75.35) | 4.14 (0.07, 446.29) | 1.10 (0.01, 176.02) | 2.13 (0.05, 143.03) | Tivantinib | 2.39 (0.04, 145.15) | 2.13 (0.03, 216.89) |
| 0.94 (0.08, 10.45) | 0.62 (0.08, 4.60) | 1.96 (0.05, 83.82) | 0.48 (0.01, 44.04) | 1.03 (0.07, 12.44) | 0.42 (0.01, 24.69) | Sunitinib | 0.92 (0.03, 21.24) |
| 1.00 (0.07, 12.77) | 0.66 (0.06, 6.03) | 1.92 (0.05, 85.26) | 0.48 (0.01, 43.19) | 1.05 (0.07, 15.80) | 0.47 (0.00, 28.64) | 1.09 (0.05, 28.68) | Sorafenib+Erlotinib |
| **DCR** |  |  |  |  |  |  |  |
| Placebo | 1.69 (0.84, 3.68) | 1.53 (0.39, 6.11) | 1.56 (0.39, 6.32) | 1.92 (0.68, 5.82) | 1.81 (0.37, 8.69) | 1.60 (0.35, 7.60) | 1.17 (0.26, 5.44) |
| 0.59 (0.27, 1.20) | Sorafenib | 0.89 (0.18, 4.15) | 0.92 (0.19, 4.25) | 1.14 (0.40, 3.20) | 1.07 (0.18, 5.95) | 0.95 (0.24, 3.65) | 0.69 (0.17, 2.62) |
| 0.65 (0.16, 2.59) | 1.12 (0.24, 5.42) | Ramucirumab | 1.01 (0.15, 6.84) | 1.27 (0.22, 7.27) | 1.18 (0.15, 9.35) | 1.06 (0.13, 8.20) | 0.77 (0.10, 5.95) |
| 0.64 (0.16, 2.56) | 1.09 (0.24, 5.28) | 0.99 (0.15, 6.76) | Everolimus | 1.23 (0.22, 7.34) | 1.18 (0.14, 9.53) | 1.04 (0.13, 8.36) | 0.75 (0.09, 5.99) |
| 0.52 (0.17, 1.48) | 0.88 (0.31, 2.52) | 0.79 (0.14, 4.49) | 0.81 (0.14, 4.46) | Brivanib | 0.95 (0.14, 6.18) | 0.84 (0.15, 4.61) | 0.62 (0.11, 3.13) |
| 0.55 (0.12, 2.69) | 0.93 (0.17, 5.51) | 0.85 (0.11, 6.87) | 0.85 (0.10, 7.03) | 1.06 (0.16, 7.36) | Tivantinib | 0.89 (0.10, 8.09) | 0.66 (0.07, 5.92) |
| 0.63 (0.13, 2.86) | 1.05 (0.27, 4.19) | 0.94 (0.12, 7.46) | 0.97 (0.12, 7.86) | 1.19 (0.22, 6.64) | 1.13 (0.12, 10.26) | Sunitinib | 0.73 (0.11, 4.89) |
| 0.85 (0.18, 3.90) | 1.44 (0.38, 5.80) | 1.29 (0.17, 9.83) | 1.33 (0.17, 10.79) | 1.61 (0.32, 9.30) | 1.52 (0.17, 13.58) | 1.37 (0.20, 9.28) | Sorafenib+Erlotinib |

Notes: SD = stable disease; PD = progressive disease; CR = complete response; DCR = disease control rate; ORR: CR +PR; DCR = SD+CR+PR; OR: odds ratio; HR: hazard ratio.
